# New approaches for detecting cancer with circulating cell-free DNA

**DOI:** 10.1186/s12916-019-1400-z

**Published:** 2019-08-16

**Authors:** Clare Fiala, Eleftherios P. Diamandis

**Affiliations:** 1 Department of Pathology and Laboratory Medicine, Mount Sinai Hospital, Toronto, Ontario Canada; 2 Department of Laboratory Medicine and Pathobiology, University of Toronto, Toronto, Ontario Canada; 3 Department of Clinical Biochemistry, Mount Sinai Hospital and University Health Network, 60 Murray St. Box 32, Floor 6, Rm L6-201, Toronto, ON MST 3L9 Canada

**Keywords:** Cell-free DNA, Early cancer diagnosis, DNA fragmentation patterns, Artificial intelligence, Cancer screening

## Introduction

Circulating cell-free DNA (cfDNA), released from normal and cancerous cells, is an exciting new biomarker. Circulating tumor DNA (ctDNA) usually contains genetic changes that could be useful for detecting cancer. Various laboratories have reported impressive clinical data, with cancer detection sensitivities ranging from 50 to 70%, calculated at 90–95% specificities [1–3].

ctDNA has additional important applications, including prognosis, monitoring therapy and estimating tumor volume [4]. These applications are less controversial than early cancer detection, and are used widely in research settings. Despite promising reports [1–3], there are some major concerns when using ctDNA for early diagnosis [5, 6].

## Sensitivity

Currently, all published investigations report sensitivities calculated from retrospectively collected samples with mixed cancer stages. The cancers were invariably diagnosed clinically or through imaging. Previously, we calculated tumor size, expected amount of ctDNA in a 10-ml blood draw, ctDNA fraction (percentage of ctDNA compared to total cfDNA; this is equivalent to mutant allele fraction, MAF), number of retrieved genomes per 10 ml of blood, and likelihood of tumor detection with ctDNA genomic analysis. We concluded that current ctDNA technologies are unlikely to detect tumors smaller than 10 mm in diameter because not enough ctDNA is retrieved for analysis (this is also known as sampling error) [5, 6]. Recent experimental data from the company GRAIL, revealed sensitivities of only 10% when testing asymptomatic breast cancer patients diagnosed with screening mammography. The sensitivity is significantly higher (44%) when testing clinically diagnosed breast cancers [7].

## Specificity

Age-related mutations have been increasingly identified in normal tissues [8]. These mutations may compromise the specificity of cfDNA analysis and lead to false positive results. Investigators usually report sensitivities at 95 or 98% specificity [1–3]. Even these seemingly high values may be insufficient when screening for less prevalent cancers, such as ovarian and pancreatic cancer. For example, a test with 100% sensitivity and 99% specificity will yield a positive predictive value (the chance of a patient having cancer if the test is positive) of only 2% if the cancer prevalence in the screened population is 1:4000 [6].

Cell-free DNA analysis cannot usually specify the affected organ/tissue in asymptomatic individuals. A positive result must be followed with costly, invasive, stressful, and potentially unsuccessful tests to identify the primary lesion. Cristiano et al. report 61% accuracy for tumor site localization, which is close to tossing a coin [3].

## A new concept for cfDNA analysis for cancer diagnosis

Recently, Cristiano et al. used cell-free DNA fragmentation length and position within the genome to diagnose cancer through low-depth genomic sequencing of multiple 5-megabase regions [3]. They discovered that cancer-derived cell-free DNA is generally shorter by about 3–6 bases, and the lengths of tumor-derived fragments are more variable than those found in controls.

Notably, instead of investigating one or a few genetic changes found only in cancer-derived DNA, they employed artificial intelligence to examine the whole spectrum length, variation and position, inferring if the pattern is cancerous. They reported sensitivities from 236 patients with various cancers ranging from 57 to 99% at 98% specificity.

## Looking closer: diagnosis of early, asymptomatic cancers

New cancer detection tests have the highest clinical value when they can identify cancer at an early, asymptomatic stage, when the chances of cure are highest. The sensitivity of Cristiano et al.’s proof-of-concept assay for detecting early stage, asymptomatic tumors (population screening) is unknown, since they only used clinically detected cancers. As demonstrated by GRAIL, sensitivity differed according to clinical versus screening detection. Sensitivity, specificity, as well as positive and negative predictive values, should be reported in future studies employing this test to account for disease prevalence.

Perhaps another way to validate these new technologies across the course of cancer diagnoses, which has not yet been widely explored, is to analyze samples collected longitudinally through randomized trials. However, early results have not yet been promising. Our group recently used such samples to evaluate and rank 49 ovarian cancer biomarkers for pre-clinical ovarian cancer diagnosis [9]. We found that none of these biomarkers were effective in detecting asymptomatic disease, and that marker performance decreased as samples were analyzed further from clinical diagnosis. Markers such as CA 125, with 80% sensitivity in detecting clinical disease, deteriorate to less than 50% sensitivity in asymptomatic patients. Future studies should use longitudinal samples for more realistic estimations of sensitivity and specificity and lead time calculation for cancer detection between asymptomatic and symptomatic stages.

## The importance of mutant allele fraction

In our previous calculations, based on experimental data, we correlated MAF to tumor volume and retrieved cancer genomes using a 10-ml blood draw [5, 6]. When there is less than one copy of a ctDNA, intermixed with 10,000 copies of normal DNA (MAF of 0.01% or less), sampling error (no retrieval of ctDNA) makes tumor detection impossible. At this MAF, the tumor will be ~ 12 mm in diameter. In most published studies, the MAF in the samples is 0.1% or higher. At this MAF, the tumor diameter will likely be more than 27 mm, and easily detectable by imaging [4, 5].

The MAF of the samples used is not mentioned in the Cristiano et al. paper; [3]. the authors only specify a MAF of < 1% in a subset of their cohort. In theory, this new method could work with less than one represented complete genome in the blood draw, since fragment length information is derived from a fraction of genomic DNA regions. However, as the MAF diminishes, the artificial intelligence algorithm’s ability to predict cancer will, predictably, progressively weaken. More data correlating MAF and sensitivity/specificity are needed.

## Conclusions

The Cristiano et al. paper demonstrates proof of concept, and does not yet comprehensively address the difficulties associated with early cancer detection. The sensitivity of this new approach and other similar methods should be confirmed in the future with experiments involving tumors of known MAF. Pre-diagnostic samples should be tested to calculate both sensitivity and lead time before clinical diagnosis, to determine if the lead time achieved with early detection using ctDNA influences patient outcomes.

## Data Availability

Not applicable.

## Abbreviations

cfDNA: cell-free DNA
ctDNA: circulating tumor DNA)
MAF: Mutant allele fraction
